# Carbon Dioxide Capture by Adsorption in a Model Hydroxy-Modified Graphene Pore

**DOI:** 10.3390/ijms241411452

**Published:** 2023-07-14

**Authors:** Paige Freyre, Emalee St. Pierre, Thomas Rybolt

**Affiliations:** Department of Chemistry and Physics, University of Tennessee at Chattanooga, Chattanooga, TN 37403, USA

**Keywords:** adsorption, binding energy, carbon dioxide, nitrogen, carbon capture, graphene, hydrogen bonding, trapping CO_2_, adsorption energy

## Abstract

Concerns regarding the environmental impact of increasing levels of anthropogenic carbon dioxide have led to a variety of studies examining solid surfaces for their ability to trap this greenhouse gas (GHG). Atmospheric or post-combustion carbon capture requires an efficient separation of carbon dioxide and nitrogen gas. We used the molecular mechanics MM3 parameter set (previously shown to provide good estimates of molecule–surface binding energies) to calculate theoretical surface binding energies for carbon dioxide ∆E(CO_2_) and nitrogen ∆E(N_2_). For efficient separation, differentiation of these two gas-surface adsorption energies is required. Examined structures based on graphene, carbon slit width pore, and carbon nanotube gave ∆E(CO_2_) to ∆E(N_2_) ratios of 1.7, 1.8, and 1.9, respectively. To enhance the CO_2_ adsorption, we developed a model graphene surface pore lined with four hydroxy groups whose orientation allowed them to form hydrogen bonds with the oxygens in CO_2_. Both the single-layer and double-layer versions of this pore gave significant enhancement in the ability to trap CO_2_ preferentially to N_2_. The two-layer version of this pore gave ∆E(CO_2_) = 73 and ∆E(N_2_) = 6.8 kJ/mol. The one- and two-layer versions of this novel pore averaged a ∆E(CO_2_) to ∆E(N_2_) ratio of 12.

## 1. Introduction

An estimated worldwide 35 billion tons (3.2 × 10^13^ kg) of carbon dioxide was emitted in 2020 [1]. With a world population of approximately 8 billion people, this means that there are roughly 4000 kg of CO_2_ emitted per person per year worldwide. Obviously, the production per person is widely distributed among the world’s population based on variables such as location, local climate, level of industrialization, conservation efforts, energy production methods, and transportation. Atmospheric increases in CO_2_ account for less than half of the total human emissions. The carbon cycle transfers carbon among the atmosphere, ocean, biosphere, and land. Ocean absorption of CO_2_ has moderated the atmospheric level but has been accompanied by ocean acidification. 

While greenhouse gases (GHGs) include methane, nitrogen oxides, and fluorinated gases, increased atmospheric carbon dioxide is the largest contributor to global warming. The burning of carbon-containing fossil fuels is the dominant source of this anthropogenic carbon dioxide [1]. 

Trapping CO_2_ is one approach to ameliorating this GHG problem. Carbon capture, utilization, and storage (CCUS) technologies require materials that can preferentially remove CO_2_ from the atmosphere or from post-combustion (air or oxygen-enriched) environments [2]. There are many practical challenges associated with gas conditions such as high temperature, humidity, impurities, and the economics of materials and renewable processes [2]. 

Although carbon dioxide is utilized naturally through the process of photosynthesis by plant life, our current environmental conditions have exceeded the capacity of natural uptake. Human activities have dramatically increased the production of greenhouse gases. These activities are varied and include areas as diverse as residential and commercial heating, transportation, electrical generation, iron and steel production, general industry, agriculture and forestry, cement manufacturing, chemical production, etc. [1].

To reduce these emissions, there is research into the process of carbon capture and storage (CCS), as well as carbon capture and utilization (CCU) for chemical or material production. There are several methods of CO_2_ capture, including optimizing the natural absorbance of carbon dioxide through plant and tree activity and natural mineralization processes. Chemical solvents can be used to separate post-combustion carbon dioxide from exhaust gas, but these processes are energy intensive [3]. 

In this work, the focus is on modeling direct carbon capture from post-combustion emissions, as for example in flue stack exhaust gases, with a focus on carbon dioxide and nitrogen gas (N_2_) separation. Our research considers a modified graphene surface model to preferentially capture carbon dioxide gas based on van der Waals forces and hydrogen bonding. 

Microporous carbons have been considered as adsorbents for carbon dioxide because of their high porosity and regeneration ease after carbon dioxide extraction [4]. The use of doped or porous graphene layers, graphene nanotubes, microporous organic polymers, metal organic frameworks (MOFs) with functional groups, and nanocrystals have been examined for a range of carbon capture compounds to find optimal conditions for adsorption [5,6,7,8,9,10]. These structures have been studied with direct experiments, as well as computational chemistry.

A literature review of carbon capturing surfaces was utilized to compare the binding energies of existing and theoretical solid structures. Within the published literature on solid-surface carbon capturing structures, very high binding energies were reported for theoretical charged boron nitride or borophene surfaces [11,12]. The structures were developed using computer simulations with the binding energies calculated through density functional theory (DFT). The CO_2_ molecule–surface binding energies for these charged pore structures ranged from 129.7 kJ/mol to 192.5 kJ/mol [11]. This binding energy dropped significantly to 17.4 kJ/mol for carbon dioxide on neutral borophene [12]. 

Although the theoretical binding energies reported were high for CO_2_ for these charged boron-containing structures, they were also high for N_2_, ranging from 65 to 79% of the CO_2_ values. In one case, the calculated N_2_ binding energy was approximately twice the CO_2_ binding energy [11]. Carbon capturing structures with applications in a nitrogen gas-rich environment must consider not only the CO_2_ binding energy but how it compares to the N_2_ value. Theoretical surfaces or pores should ideally enhance carbon dioxide adsorption while simultaneously not enhancing nitrogen gas adsorption. In the remainder of this work, only overall neutral surfaces were considered. 

Metal organic frameworks (MOFs) or unfunctionalized metallic linkers are an example of carbon capturing structures, where graphene sheets are doped with inorganic metal atoms or metal-based functional groups. The binding energies for these structures varied from 13.4 kJ/mol to 61.5 kJ/mol [10,13,14,15]. Reported values were determined using different methods, including quantum chemical methods, DFT, and experimental determinations. The experimental binding energies were 49.3 kJ/mol and 45 kJ/mol, both of which fall within the range established from the calculated values for MOFs [16,17]. 

Reported studies have also explored a variety of carbonaceous structures, including carbon nanotubes, ultra-microporous carbons, activated carbon, and graphene structures [17,18,19,20,21,22,23,24,25,26]. The reviewed articles used different methods for determining the binding energy, including DFT, grand canonical Monte Carlo (GCMC) simulations, and experiments such as thermal desorption spectroscopy (TDS). The reported binding energies ranged from 65.1 kJ/mol for single-walled carbon nanotubes to 15.2 kJ/mol for single-layer graphene [18,26]. 

Table 1 provides a summation of the reported binding energies for carbon dioxide. The range of the binding energies found in Table 1 is 13.4 to 65.1 kJ/mol on neutral carbon-based structures and MOFs. While most structures did not examine the binding energy for nitrogen gas on their surface, the differences, where reported, were not highly selective with respect to CO_2_ and N_2_. Binding energies for both CO_2_ and N_2_ were reported for pyrolytic graphite, activated carbon, and carbon nanotube, with CO_2_ providing 24, 27.1, and 65.1 kJ/mol and with N_2_ providing 13, 12.8, and 50.5 kJ/mol, respectively [17,18,22]. Therefore, these values give carbon dioxide to nitrogen binding energy ratios of 24/13 = 1.8 for pyrolytic graphite, 27.1/12.8 = 2.1 for activated carbon, and 65.1/50.5 = 1.3 for a carbon nanotube. This lack of differentiation of carbon dioxide and nitrogen gas binding energies makes it difficult to separate these molecules.

For considering an application to exhaust gases, this research compared the binding energy from the capture of the GHG, carbon dioxide, and the benign counterpart, nitrogen gas, from which it must be separated. Our research explored the binding energies of carbon dioxide and nitrogen molecules with a modified graphene surface pore containing four hydroxy groups. Our goal was to create a model surface that produces a significant enhancement of the CO_2_ attraction with little corresponding increase in the N_2_ attraction. We present a structure that uses hydrogen bonding to selectively enhance CO_2_ binding energy and provides for a systematic variation of binding energy based on the organization of hydroxy groups within this graphene pore. 

## 2. Results

### 2.1. One-Layer and Two-Layer Graphene Structures

In prior work, we effectively have used molecular mechanics (MM) to calculate molecule–surface binding energies (∆E) [26,27]. Comparing calculated ∆E values using molecular mechanics and experimental molecule–surface binding energies has supported the use of molecular mechanics for modeling interactions on carbon surfaces [26,27]. In this work, we use molecular mechanics with the MM3 parameter set [27]. 

Prior to the pore creation, two starting structures were used: a 300 carbon atom flat, single-layer graphene model; a 600 carbon atom flat, two-layer graphene model. In the two-layer model, the graphene layers are offset as in Bernal graphite. The optimized surface structures were found and then locked, and a single gas molecule was systematically placed near the center of each graphene surface in six different orientations over the bonds, rings, and atoms of graphene with each placement repeated three times. The molecule–surface binding energy was calculated for each orientation and used to determine the average binding energy (∆E) and standard deviation for carbon dioxide and nitrogen molecules on both the single-layer and double-layer graphene surfaces. 

The average of the 18 ∆E measurements (and standard deviation) for each molecule on the single graphene surface gave for CO_2_ 13.8 (±0.06) and N_2_ 8.3 (±0.04) kJ/mol. The average ∆E on the two-layer graphene was found to be for CO_2_ 15.0 (±0.07) and N_2_ 8.8 (±0.18) kJ/mol. The standard deviation as a percentage of the corresponding average ∆E ranged from 0.4 to 2.0%. Repeated measurements of ∆E show little variation, which leads to the conclusion that the attraction of the gas molecules to the graphene sheets are fairly uniform despite orientation. When a gas like carbon dioxide or nitrogen gas is moved across a uniform graphene surface, the exact placement of the molecule on the surface will have only a small effect on the overall binding energy. 

A separation of CO_2_ from N_2_ gas requires a differentiation of the binding energies between the two molecules. A more effective separation of the molecules requires a larger difference in the ratio of their binding energies. The binding energy ratio for the single graphene layer is ∆E(CO_2_)/∆E(N_2_) = 13.8/8.3 or 1.66. The binding energy ratio for a double graphene layer is ∆E(CO_2_)/∆E(N_2_) = 15.0/8.8 or 1.70. Due to the similarity in size of the molecules, the van der Waals (vdW) forces are similar for the two molecules in their attraction to graphene carbon surfaces, and as a result their binding energy ratio is less than two. Our goal is to create a model surface structure that can bind CO_2_ effectively while at the same time significantly enhancing the CO_2_ to N_2_ binding energy ratio.

Prior experimental values for the molecule–graphite binding energy were compared to the results from these molecular mechanics calculations using the MM3 parameter set. The experimental values previously found with gas–solid chromatography were based on retention time determinations over a range of temperatures and the corresponding van’t Hoff plots. In this prior work, the experimental gas–graphite surface binding energy (∆E) was found to be ∆E(CO_2_) = 17.3 and ∆E(N_2_) = 9.2 kJ/mol or ∆E(CO_2_) = 17.2 kJ/mol [28,29]. Experimental values of ∆E are similar to our calculated two-layer values of 15.0 kJ/mol for CO_2_ and 8.8 kJ/mol for N_2_ agreeing within better than 15% error for CO_2_ and within 5% error for N_2_. In addition, the experimental ratio of ∆E(CO_2_)/∆E(N_2_) ratio is 1.9, which is similar to our 1.7 computed result. As the experimental graphite surface is not completely flat, it is not surprising that the experimental values for carbon dioxide and nitrogen gas were larger than our idealized two-layer model graphene surface.

In our calculations, we also observed a two-layer to one-layer binding energy ratio of 1.09 and 1.06 for CO_2_ and N_2_, respectively. This agrees with past observations that a second graphene layer adds less than 10% to the molecule–surface binding energy from a single graphene layer. And a third layer contributes only about 1%. This observation means that two layers of graphene may be considered a reasonable model for multilayer graphite vdW adsorption [26]. 

### 2.2. Pore Structure with Four Hydroxy Groups

**Figure 1:**
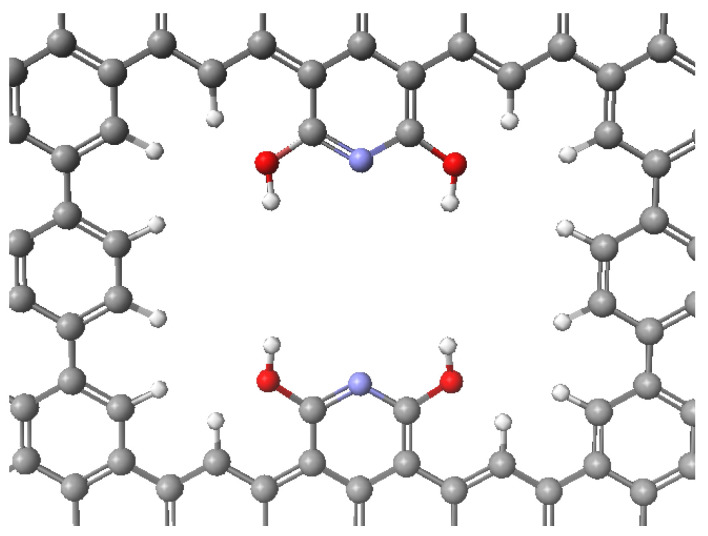
Four hydroxy graphene pore structure. Atom colors: carbon—gray; hydrogen—white; nitrogen—purple; oxygen—red.

**Figure 2:**
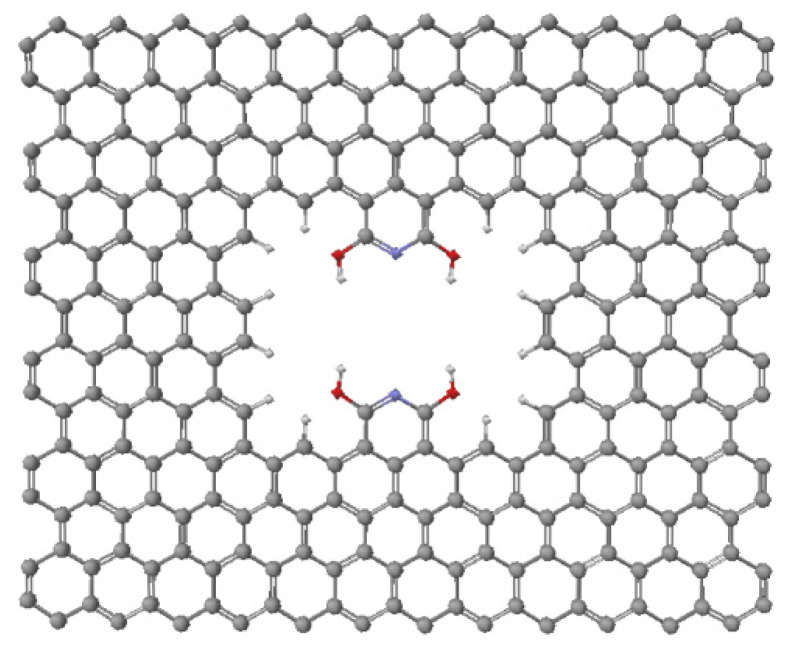
Optimized pore in one-layer graphene. Atom colors: carbon—gray; hydrogen—white; nitrogen—purple; oxygen—red.

Figure 1 shows the pore structure we developed in this work in order to increase the differentiation of carbon dioxide and nitrogen binding energies. This pore was placed in the center of the graphene layer as shown in Figure 2. We anticipated the formation of hydrogen bonds between the hydroxy OH groups within the pore and the oxygens in CO_2_.

As a single layer, the pore layer contains 290 atoms (Figure 2). However, if this pore layer is placed on top of a graphene layer described previously, then the two-layer pore contains 590 total atoms. That is a 300 carbon atoms bottom graphene layer and a 290 atoms top layer that contains the pore. The pore consists of a perimeter lined with hydrogen atoms bonded to carbon atoms (12 C-H groups), two nitrogen atoms, and four hydroxy groups. The presence of the two nitrogen atoms within the pore provides more room than two CH groups previously present in the graphene layer. This change from CH to N provides more space for an adsorbed CO_2_ molecule to fit lower in the pore. 

An examination of the torsional rotation energy associated with a single N-C-O-H dihedral angle showed a significantly lower energy for the orientation with the OH pointed toward the center of the pore as compared to its counterpart with the OH pointed back toward the perimeter of the pore. There is a difference of 69 kJ/mol between the more stable N-C-O-H dihedral angle of zero degrees (OH pointed toward the middle of the pore) and the less stable N-C-O-H dihedral angle of 180 degrees (OH pointed back toward the perimeter of the pore). The 180-degree orientation represents the significant steric hindrance. This structure (Figure 1) ensures that the four OH groups will point toward the middle of the pore where they will be available to form hydrogen bonds with the oxygens in the CO_2_ molecule. 

**Figure 3:**
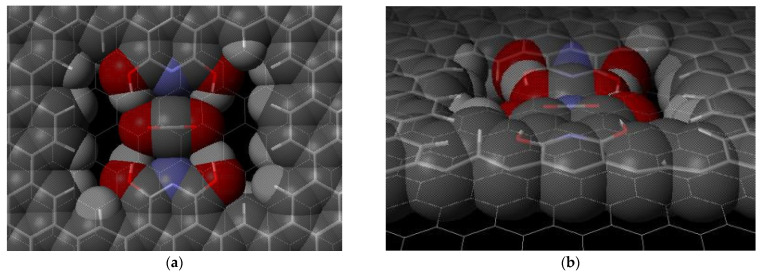
Carbon dioxide in a two-layer pore structure, optimized geometry with view from (**a**) above and (**b**) side. Lines show bonds in top and bottom graphene layers. Atom colors: carbon—gray; hydrogen—white; nitrogen—purple; oxygen—red.

**Figure 4:**
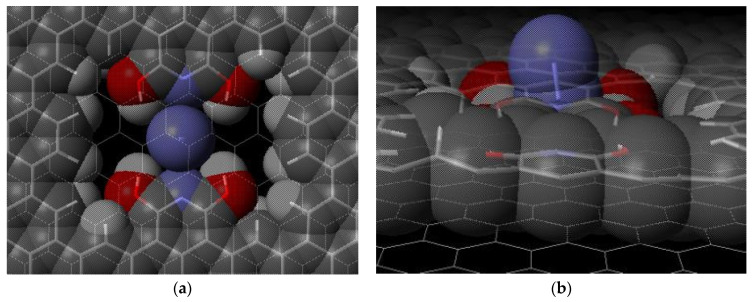
Nitrogen gas in two-layer pore structure, optimized geometry with view from (**a**) above and (**b**) side. Lines show bonds in top and bottom graphene layers. Atom colors: carbon—gray; hydrogen—white; nitrogen—purple; oxygen—red.

A CO_2_ molecule or a N_2_ molecule was individually placed parallel or perpendicular within the pore region and slightly above the plane. The molecules are pulled into place in the pore. The CO_2_ favors a parallel orientation (Figure 3), while the N_2_ favors a perpendicular orientation (Figure 4) with respect to the plane of the surface. These favored orientations occur regardless of the initial orientation of the molecules. The ∆E values were determined using Equation (2). The E_ms_ values represent the carbon dioxide or nitrogen gas molecule in an optimized pore location. The isolated molecule energy E_m_ and isolated surface energy E_s_ are combined (E_m_ + E_s_), and the difference of this value and E_ms_ gives the binding energy, ∆E. 

The hydrogens that line the perimeter of the pore and the hydroxy groups were unlocked and free to move while the surrounding graphene surface was fixed in place. The hydrogen atoms and hydroxy groups must be free to move to achieve maximum stabilization of the molecule in the pore. The molecule capture results for carbon dioxide were for the two-layer pore ∆E(CO_2_) = 73 kJ/mol and the one-layer pore ∆E(CO_2_) = 71 kJ/mol. For nitrogen, the two-layer results were ∆E(N_2_) = 6.8 kJ/mol and for one layer ∆E(N_2_) = 5.4 kJ/mol.

### 2.3. Variation of the Number of Hydroxy Groups 

**Figure 5:**
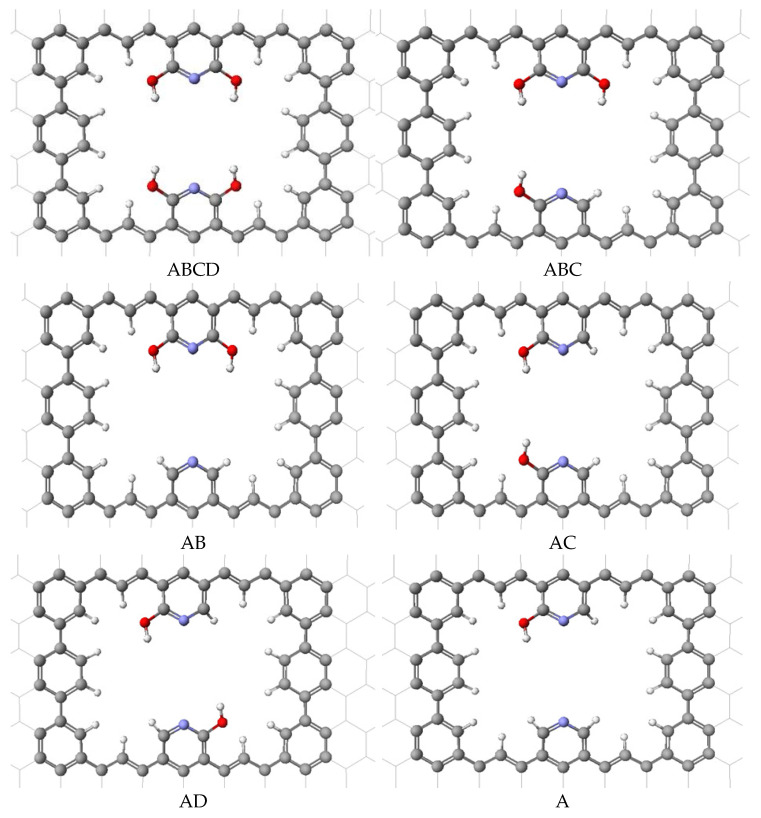
Arrangements of the substructure surfaces used to calculate binding energies for carbon dioxide and nitrogen. Atom colors: carbon—gray; hydrogen—white; nitrogen—purple; oxygen—red.

From this primary pore structure (Figure 1), we identified six possible substructures with varying numbers and locations of hydroxy groups, from one to four. With the one-layer pore structure, we calculated the binding energies for carbon dioxide and nitrogen gas individually within each possible pore. We denoted each hydroxy group location with A, B, C, or D, from top left, top right, bottom left, and bottom right, respectively (Figure 5). 

For all six structures, the molecule of interest was initially placed on the surface in a parallel orientation with respect to the surface. An optimized geometry experiment was performed for each molecule on each surface to determine the binding energy of the molecules using Equation (2) as described previously. MM3 ∆E computations were carried out for carbon dioxide and nitrogen on the six different surface pores shown in Figure 5. The results are summarized in Table 2. 

From this data, we see that the nitrogen gas remained relatively unchanged from four hydroxy groups to one hydroxy group. For nitrogen, the ∆E(N_2_) values were in the range of 5 to 7 kJ/mol. This small variation of ∆E, as the OH groups were changed, was due to a lack of hydrogen bonding with nitrogen. However, for the carbon dioxide, as the number of hydroxy groups increased, then ∆E(CO_2_) increased from 29 kJ/mol to 70 kJ/mol, meaning an increase in the adsorption capabilities of the pore. These results demonstrate how the hydroxy groups’ orientation toward the center of the pore was suitable for the formation of hydrogen bonds with both oxygens in CO_2_ but not with a nitrogen in N_2_. This geometry affects the preferential nature of the pore for carbon dioxide gas over nitrogen gas. In addition, this variation in the number of OH groups can be used to adjust the binding energy for the adsorption of carbon dioxide.

For the AB, AC, and AD surfaces, the two OH groups are essentially the same binding energy within the uncertainty of repeated measurements. In other words, the CO_2_ is able to form two hydrogen bonds effectively even as the position of the OHs are varied within the pore. However, the variation in the total number of hydroxy groups is able to adjust the strength of binding for CO_2_ while leaving the N_2_ essentially unchanged. Thus, the binding energy ratio ∆E(CO_2_)/∆E(N_2_) can be adjusted over a range of values with a high and low ratio of 13 and 4, respectively. The pore size is such that neither molecule will pass through the single-layer pore. The molecules will be attracted to the surface but will not be able to pass through the single-layer pore because of the size of the molecule relative to the pore opening. 

### 2.4. Comparison to Other Model Carbon Surfaces 

To compare our single-layer pore to other single-layer carbon surfaces, we used several single-walled carbon nanotubes of varying diameters and also a series of slit pores of varying diameter to compare ∆E values among these model surfaces. 

We used three single-walled carbon nanotubes (CNTs) containing 18 hexagonal rings along the length of each CNT with (5,5), (8,8), and (9,9) armchair CNT structures and diameters 0.7, 1.1, and 1.2 nm, respectively. For each CNT a carbon dioxide or nitrogen molecule was placed in the center of the CNT with the linear portion of the molecule parallel to the nanotube length axis. 

The binding energies for the molecule in the CNT were found relative to an isolated molecule and isolated CNT using Equation (2). Binding energy values found for the (5,5), (8,8), and (9,9) CNTs were 37, 25, and 23 kJ/mol for CO_2_ and were 19, 15, and 14 kJ/mol for N_2_, respectively. As the CNT diameter was increased, binding energies decreased, and the ∆E(CO_2_)/∆E(N_2_) binding energy ratio decreased from 1.9 to 1.7 to 1.6. Like the graphite surface, CNTs do not give significant differentiation between CO_2_ and N_2_ molecule–surface interactions, even for the small diameter (5,5) CNT. 

We created a slit pore using two parallel 300 atom graphene sheets and varied the graphene separation to model various diameter pores. Using our slit pore, we calculated the binding energy for pore widths ranging from 0.60 nm to 1.44 nm. Molecules of interest were placed directly in the center of the pore. 

Slit pores were created using two parallel single-layer graphene sheets at inter-nuclei separations of 0.60, 0.65, 0.70, 0.75, 0.80, 0.85, 0.90, 1.25, and 1.45 nm. These nine pores were used to calculate binding energies for CO_2_ and N_2_ placed in the center of each pore. As expected the binding energy increased as the pore diameter was decreased until a maximum ∆E value was achieved when the two walls were such that molecule barely fits in the pore. As the pore was made still narrower from the optimum diameter, then the binding energy decreased. If the pore is too narrow, then the molecule–pore interaction becomes repulsive as the pore is too small for the molecule to fit. Our results showed an optimal slit width between 0.65 to 0.70 nm with ∆E of about 28 kJ/mol (0.29 eV) for carbon dioxide within the pore and about 16 kJ/mol (0.17 eV) for nitrogen within the pore. These results give around a 1.7 ∆E(CO_2_)/∆E(N_2_) binding energy ratio. The binding energy results for CO_2_ and N_2_ and the corresponding energy ratios are summarized in Table 3.

In prior work, Kwac et al. used density functional theory with a dispersion correction (DFT-D2) within the Vienna ab initio Simulation Package (VASP) to calculate ∆E values [25]. Our binding energies were compared to these prior results in support of the utility of molecular mechanics MM3 calculations for molecule–surface interactions. For slit width carbon pore models that varied over a range of separation, Kwac reported the optimized slit widths to be 0.62 nm with ∆E(CO_2_) approximately 0.30 eV and 0.70 nm with ∆E(N_2_) approximately 0.20 eV [25]. These results compare well to our values of 0.29 and 0.17 eV for CO_2_ and N_2_, respectively. Another common trend is seen in that as the slit width decreased, the binding energy increased. Their reported optimal pores were 0.62 nm for CO_2_ and 0.70 nm for N_2_. These results are similar to our optimal slit width between 0.65 to 0.70 nm for both CO_2_ and N_2_.

In Kwac’s work, different molecule–graphene sheet distances and binding energies were reported for CO_2_ and N_2_ in different positions relative to a carbon surface structure [25]. They used a 32-carbon atoms structure as a model for a graphene sheet. They placed the carbon dioxide and nitrogen molecules on the structures in various positions. We recreated their structure and placed molecules in the same orientation as their parallel structures. We ran optimized MM3 geometry calculations in triplicate on these structures and noted the average optimized distances and energies. Their reported binding energies for CO_2_ and N_2_ were equivalent to 15.2 and 10.5 kJ/mol, respectively. Recreating their molecule and surface positions when we optimized the geometry, we found 13.9 and 9.7 kJ/mol. Their molecule–surface internuclei separations were 0.32 nm and 0.33 nm, and ours were 0.34 and 0.35 nm for CO_2_ and N_2_, respectively. 

Our results with MM3 molecular mechanics for molecule–surface energy interactions provided a good match to this prior work based on a DFT-D2 computational approach. Two other DFT calculations reported ∆E(CO_2_) on a single-walled carbon nanotube and on a carbon nanotube bundle with values of 14.2 and 14.6 kJ/mol, respectively [30,31]. These results are similar to Kwac’s ∆E(CO_2_) on graphene calculation of 15.2 kJ/mol and our own value of 13.9 kJ/mol.

## 3. Discussion

A summary of our calculated binding energies for various surface structures is given in Table 3. From these results, it is clear that transitioning from a flat graphitic surface to either a slit pore structure or a small diameter carbon nanotube gives an enhancement of molecule capture binding energies but only little improvement in the ability to differentiate between CO_2_ and N_2_ binding energies. The carbon dioxide to nitrogen binding energy ratio, ∆E(CO_2_)/∆E(N_2_), for a graphitic surface is about 1.7 and changes for the best slit pore to a ratio of 1.8 (Table 3). For a (5,5) CNT this binding energy ratio is about 1.9. However, with the four hydroxy pore structures, there is a more than ten-fold enhancement of the binding energy for CO_2_ relative to N_2_, with one layer about 13 and the two layer about 11. In both cases, the nitrogen binding energy remains low while the carbon dioxide is significantly enhanced relative to a regular graphene layer. 

In Table 3, ∆E(CO_2_) values are 14, 28, and 37 kJ/mol for one-layer graphene, a 0.68 nm slit pore, and a (5,5) CNT, respectively. This enhancement is expected as additional carbon surface is effectively brought into proximity with the CO_2_ molecule. In going from a single graphene layer to two layers with the molecule between, ∆E(CO_2_) is doubled from 14 to 28 kJ/mol. In the carbon nanotube, the small CO_2_ molecule becomes surrounded by still more carbon surface than the slit pore, the vdW interaction is further increased, and ∆E(CO_2_) increases by approximately 32% going from 28 to 37 kJ/mol. 

A similar pattern is observed for ∆E(N_2_) with an increase from roughly 8 to 16 kJ/mol from one-layer graphene to a 0.68 nm slit pore. For the slit pore to CNT change, there is a 19% increase in ∆E(N_2_) as it changes from 16 to 19 kJ/mol. A similar pattern is observed for these two small molecules in their vdW surface attraction enhancements as the carbon surface structure is changed. However, given the similarity of ∆E increases, there is no significant enhancement in the ability of the carbon slit pore or the CNT to favor the adsorption of one molecule over the other. However, we observe a considerable enhancement of ∆E(CO_2_) relative to ∆E(N_2_) in both the single-layer four hydroxy pore and the double-layer four hydroxy pore (Table 3). In these designed pores, the ∆E(CO_2_) to ∆E(N_2_) ratio favors CO_2_ adsorption by a factor of about 12 ± 1. 

A review of the literature for solid-surface models and experimental values of ∆E revealed a wide range of reported binding energy values for structures used to trap carbon dioxide with varying functional groups, pore sizes, and pore structures. Table 1 provides a summary of these reported binding energies for carbon dioxide. The ∆E(CO_2_) values span a range from 13.4 to 65.1 kJ/mol. It has been previously shown that some DFT binding energy calculations based on vdW forces do not agree with experimental values unless a dispersion correction is included [26]. 

Our two-layer model structure had a carbon dioxide binding energy of 73 kJ/mol. Our model structure utilizes the formation of four hydrogen bonds with the two oxygens of carbon dioxide molecule to trap the molecule. The optimized interaction is shown in Figure 3. The CO_2_ parallel orientation allows for maximum interaction and binding energy. Figure 4 shows the favored geometry for a nitrogen molecule within the pore structure to be in a perpendicular orientation. Our binding energy for carbon dioxide exceeds the twenty reported values in Table 1 and demonstrates how potentially effective the proposed or similar structure may be at capturing carbon dioxide. This research explores the change in binding energy for a carbon dioxide molecule compared to a nitrogen molecule on our proposed structure (Figure 1) and the unique sensitivity of the four hydroxy graphene pore as a theoretical carbon capture surface. 

This research demonstrated a systematic selectivity within the designed pore by varying the number of hydroxy functional groups within the pore. Varying the number of hydroxy groups was able to adjust the carbon dioxide binding energy as shown in Table 2. Each additional hydroxy group adds about 13 to 15 kJ/mol of carbon dioxide–pore interaction energy. However, the nitrogen–pore binding energy remains fairly constant even as the number of hydroxy groups is varied. 

We developed a model graphene-based carbon capturing structure designed with four hydroxy groups lining a pore, providing four hydrogen bonding sites, and creating a distinct selectivity for carbon dioxide over nitrogen gas. Our optimized two-layer and one-layer pore structures had molecule binding energies that significantly favored CO_2_ over N_2_ by an energy ratio of about 12 to 1. Since energy differences relate to equilibrium distribution as an exponential factor, these binding energy differences would provide for a considerable enhancement of CO_2_ trapping relative to N_2_. 

Although application would likely require the pre-removal of water molecules and the practical synthesis of an achievable surface or membrane, the role of hydrogen bonding for preferentially trapping CO_2_ gas should continue to be explored. In addition, separating carbon dioxide from other post-combustion gases or from the air will be an important process, either permanently trapping CO_2_ for carbon capture and storage (CCS) or utilizing it as a feedstock for chemical production through carbon capture and utilization (CCU). 

## 4. Materials and Methods

To calculate the energies for our surfaces, molecules, and molecule–surface structures, we employed computational chemistry using Scigress version 7.7.0.49 (Fujitsu, Tokyo, Japan) implementing molecular mechanics with Allinger’s MM3 parameter set [26,27]. Molecular mechanics is based on laws of motion to determine atomic forces and adjust positions and orientations of atomic nuclei with respect to each other to find the optimal or lowest steric energy [28]. Molecular mechanics does not depend on electronic wave functions or electron densities but is based on energy equations using optimized parameters [28]. Within Scigress calculations for optimizing geometry using MM3 parameters, the sum of all forces results in a steric or potential energy, which represents the calculated energy of a molecular or surface structure where the energy is at a minimum. 

The forces acting on a chemical sample are a summation of the atomic positions representing bond stretch, bond angle, stretch bend, dihedral angle, improper torsion, van der Waals, electrostatics, hydrogen bonds, torsion stretch, and bend–bend interactions. Likewise, the total energy, E, of a collection of interconnected atoms is given by the sum of all of the individual atom–atom stretches, atom–atom–atom angles, and so forth, for all of the covalent terms along with the noncovalent terms related to hydrogen bonds, van der Waals forces, and electrostatics, as shown below

(1) $$E=E_{stretch}+E_{angle}+E_{stretchbend}+E_{dihedralangle}+E_{impropertorsion}+E_{vdW}+\;E_{electrostatic}+E_{hydrogenbonds}+E_{torsionstretch}+E_{bend-bendinteractions}.$$

Each of the component energy contributions, such as bond stretching, has a functional form and is fitted with empirical parameters based on the specific atoms involved [26]. The bond stretching energy parameters for a given bond, to take as one example, are based on experimental data, such as X-ray diffraction, infrared spectroscopic, or thermodynamic bond energies [27]. 

Calculations based on force field parameters cannot provide electronic details or predict properties that may require electron structure calculations. However, molecular mechanics can provide the relative energies of different molecular conformations and atomic structures and gas–surface interactions where noncovalent interactions dominate. In the present work this binding energy is largely dependent on van der Waals (vdW) forces and hydrogen bonding. Molecular mechanics have the advantage that calculations can be quickly done for systems with thousands of atoms [26]. 

The steric energy varies as different three-dimensional atomic positions are examined using molecular mechanics until the lowest steric energy conformation is identified. Molecular mechanics optimization techniques find an optimal conformation by adjusting atomic positions until the lowering of the steric energy is negligible. In order to calculate the molecule–surface binding energy (ΔE) with the MM3 parameter set, the geometry is optimized to determine the energy for a carbon dioxide molecule or a nitrogen gas molecule. The lowest reported molecule steric energy is labeled E_m_. An optimized geometry experiment is then performed on a surface structure, and the lowest steric energy is reported as E_s_. The E_m_ and E_s_ are summed to represent the total energy of an isolated molecule and isolated surface with no interactions between them. 

A molecule is then placed on or near the surface at a specific orientation and distance, and an optimized geometry calculation is performed to determine the lowest steric energy of the molecule–surface structure, E_ms_. This value represents the energy of the molecule and structure interacting, for example, by van der Waals forces. 

The molecule–surface binding energy, ΔE, represents the energy required to remove the molecule from the surface and is given by

ΔE = (E_m_ + E_s_) − E_ms_. (2)

The more positive this value, the stronger the molecule–surface interaction energy and, therefore, the more efficient a molecular capturing structure. The less positive the value, the weaker the bonding or stronger the repelling force between the molecule and structure. If the order of subtraction in Equation (2) is reversed, then negative values represent the energy released as the molecule binds to the surface. Then, the more negative the value, the stronger the attraction and, therefore, the more efficient the capturing structure. For convenience, we use Equation (2) and report the favorable molecule–surface stabilization interactions as positive values.

In prior work, experimental binding energies for 10 alkanes and halogenated alkanes were determined [26]. Values were found from gas–solid chromatography with temperature variation and van’t Hoff plots. As the model was made more appropriate for the porous carbon surface, the fit between experimental and theoretical values improved. With the best model, the slope of experimental versus calculated binding energies gave a slope of 1.025 with an R^2^ of 0.978. The difference between the experimental and calculated ∆E values provided a relative standard deviation of 4.4%. The MM2 and MM3 parameter sets are able to provide good estimates of ∆E. Therefore, we might expect a ∆E error in the current work of 5% or higher.

Compared to the commonly used computational method of DFT, MM3 does not consider electronic properties. However, DFT often uses a dispersion correction calculation sometimes denoted as DFT-D or vdW-DFT, to achieve computations that are representative of experimental data. There has been much previous research to justify the use of molecular mechanics MM2 and MM3 parameters that have shown to provide good molecule–carbon surface binding energies compared to experimental data based on noncovalent interactions such as vdW and hydrogen bonding [26]. 

In order to further validate the use of MM3 as an appropriate parameter set for our research, we first performed experiments to calculate the binding energies of carbon dioxide and nitrogen gas on single-layer and double-layer graphene and compared them to experimental values. For the limited purpose of providing calculated ∆E values based primarily on vdW and hydrogen bonding that are suitable approximations for experimental values, molecular mechanics can serve this purpose well. The MM2 and MM3 parameter sets have been used effectively to estimate noncovalent interactions based on vdW and hydrogen bonding interactions [26]. 

## Figures and Tables

**Table 1:** Calculated and experimental carbon dioxide surface binding energy ∆E(CO_2_) values from different references (Ref.).

Ref.	Structure Type	Structure	Method	∆E(CO_2_) (kJ/mol)
[18]	carbon	carbon nanotube	vdW-DFT	65.1
[13]	MOF	functionalized MOF	QC method ^1^	61.5
[16]	MOF	functionalized MOF	NLDFT ^2^	49.3
[17]	silicate	Zeolite	experimental	45
[19]	carbon	MWCNT ^3^	experimental	32
[20]	carbon	ultra-microporous	experimental	27.2
[17]	carbon	activated carbons	GCMC simulation	27.1
[19]	carbon	activated carbons	experimental	27
[14]	MOF	aluminum nitride	DFT	26.1
[21]	carbon	organic polymers	DFT	24
[22]	carbon	pyrolytic graphite	experimental TDS ^4^	24
[23]	carbon	CNT bundle	experimental	22.5
[24]	carbon	activated carbon	experimental	21
[15]	MOF	functionalized MOF	DFT	20.5
[25]	carbon	graphene	DFT	18.3
[12]	boron	neutral borophene	DFT	17.4
[15]	MOF	functionalized MOF	DFT	16.5
[26]	carbon	graphene	DFT	15.2
[13]	linkers	functionalized	quasicontinuum	13.5
[15]	MOF	functionalized MOF	DFT	13.4

^1^ Quantum chemical. ^2^ Nonlocal DFT. ^3^ Multiwalled carbon nanotube. ^4^ Thermal desorption spectroscopy.

**Table 2:** Molecule–surface binding energies on the graphene hydroxy one-layer pore for A (one hydroxy); AB, AC, and AD (two hydroxy); ABC (three hydroxy); and ABCD (four hydroxy) as shown in Figure 5.

Surface	∆E(CO_2_)	∆E(N_2_)	Energy Ratio
Hydroxy Groups	(kJ/mol)	(kJ/mol)	∆E(CO_2_)/∆E(N_2_)
ABCD	70.3	5.4	13
ABC	56.5	5.0	11
AB	42.3	5.0	8.5
AC	41.8	5.4	7.7
AD	42.3	5.4	7.8
A	28.9	7.1	4.1

**Table 3:** Calculated molecule–surface binding energies and energy ratios with graphene, carbon nanotube, slit pore 0.68 nm diameter, and four hydroxy pore structure. One- and two-layer hydroxy pores show significant enhancement of CO_2_ binding energy relative to N_2_.

Surface	∆E(CO_2_) (kJ/mol)	∆E(N_2_) (kJ/mol)	Energy Ratio ∆E(CO_2_)/∆E(N_2_)
Graphene—one layer	13.8	8.3	1.7
Graphene—two layer	15	8.8	1.7
(5,5) CNT	37	19	1.9
Slit pore 0.68 nm	28	16	1.8
Hydroxy pore—one layer	71	5.4	13
Hydroxy pore—two layer	73	6.8	11

## Data Availability

Additional data may be obtained from T.R.
